# Finding the subitizing in groupitizing: Evidence for parallel subitizing of dots and groups in grouped arrays

**DOI:** 10.3758/s13423-021-02015-7

**Published:** 2021-10-20

**Authors:** Theresa E. Wege, Kelly Trezise, Matthew Inglis

**Affiliations:** Centre for Mathematical Cognition, Loughborough University, Epinal Way, Loughborough, Leicestershire, LE11 3TU UK

**Keywords:** Enumeration, Visual grouping, Subitizing

## Abstract

‘Groupitizing’ refers to the observation that visually grouped arrays can be accurately enumerated much faster than can unstructured arrays. Previous research suggests that visual grouping allows participants to draw on arithmetic abilities and possibly use mental calculations to enumerate grouped arrays quickly and accurately. Here, we address how subitizing might be involved in finding the operands for mental calculations in grouped dot arrays. We investigated whether participants can use multiple subitizing processes to enumerate both the number of dots and the number of groups in a grouped array. We found that these multiple subitizing processes can take place within 150 ms and that dots and groups seem to be subitized in parallel and with equal priority. Implications for research on mechanisms of groupitizing are discussed.

We normally do not need to count in order to enumerate sets of just a few items. The fast and accurate process of enumerating small sets is known as subitizing and is distinct from counting (Choo & Franconeri, 2014; Jensen et al., 1950; Trick & Pylyshyn, 1994). The maximum number of items that can be enumerated without counting within a fraction of a second is known as the subitizing range, and typically extends to four items (Leibovich-Raveh et al., 2018; Simons & Langheinrich, 1982; Trick & Pylyshyn, 1994). To exactly enumerate a set of more items, we might need to slowly and effortfully count one by one. This changes if items in a large set are grouped into smaller sets. Enumeration of large sets in grouped arrays can be facilitated to be fast and accurate, similar to the subitizing of small sets. This phenomenon of facilitated enumeration in grouped arrays is known as ‘groupitizing’ (Starkey & McCandliss, 2014). The mechanism behind groupitizing is still a matter of debate. Various forms of mental calculation have been proposed as a mechanism, but it is unclear how exactly the operands for those calculations are found. In this paper, we investigate the possibility that grouping a large set of dots into a small number of groups allows subitizing of both the number of dots in each group and the number of groups. We further investigate whether these multiple subitizing processes are organized sequentially or happen in parallel. We then discuss how multiple subitizing processes in grouped arrays can be a foundation of groupitizing mechanisms by providing the operands for mental calculations.

## Subitizing in ‘groupitizing’

A central idea in many groupitizing studies is that grouped arrays can be enumerated faster than unstructured arrays, because the visually grouping of dots into smaller sets allows participants to use mental calculation, such as adding up of the numbers of dots in each group (Anobile et al., 2020; Moscoso et al., 2020; Wender & Rothkegel, 2000) or multiplying the number of dots per group by the number of groups (Ciccione & Dehaene, 2020). Finding the operands for such mental calculations is a matter of enumerating small sets quickly and accurately. In other words, groupitizing mechanisms seem to require input from subitizing dots and groups in grouped arrays.

Subitizing as a foundation for groupitizing has to date only been discussed in terms of subitizing the number of dots in each group: one group after another (Anobile et al., 2020) or in terms of an extended subitizing range (Moscoso et al., 2020). Both possibilities limit subitizing in grouped arrays to subitizing the number of dots. However, subitizing can occur regardless of an item being a visually separate entity (such as a single dot) and occur simply because an item is perceived as an entity (such as a group of dots; Chesney & Haladjian, 2011; Pagano & Mazza, 2012; Piazza et al., 2011; Porter et al., 2016). Visual grouping might support groupitizing by creating not only small subitizable groups of dots but also by potentially creating a subitizable number of groups. This is particularly apparent for groupitizing via mental multiplication. Ciccione and Dehaene (2020) found that the groupitizing effect is most pronounced in grouped arrays with a small number of identical groups containing a small number of dots. Participants seem to enumerate such arrays by multiplying the number of dots by the number of groups. Finding the operands required for this groupitizing mechanism might be best explained by multiple subitizing processes that are supported by visual grouping.

## Subitizing dots and groups in grouped arrays

In order to test the assumption that groupitizing is supported by multiple subitizing processes, we need to assess whether it is possible for participants to subitize both the number of dots and groups in a grouped array. Subitizing is characterized by being fast and accurate. Its success can be tested by presenting grouped arrays for a fraction of a second (~150 ms) and measuring whether participants could accurately enumerate both dots and groups in the array (for a similar methodology, see Melcher et al., 2020).

If participants show successful subitizing of both dots and groups, the question follows how these multiple subitizing processes are organized. Are they organized sequentially, with one subitizing process being prioritized, or do they happen in parallel with equal priority? Friedenberg and Limratana (2005) investigated multiple enumeration processes of estimation by asking participants to estimate both the number of groups and the number of dots in grouped arrays. Their methodology and findings provide us with a framework of interference effects between multiple enumeration processes that can be used to disentangle patterns of sequential versus parallel organization of such processes. Interference between the enumeration of dots and groups was measured as the systematic overestimation/underestimation that one enumeration process caused in the other (on systematic underestimation of grouped arrays, see Allik & Tuulmets, 1991; Bertamini et al., 2016; Chesney & Gelman, 2012; Ginsburg, 1976; Im et al., 2016). Sequential enumeration processes should be characterized by a stronger interference of the prioritized enumeration process with the other enumeration process than vice versa (i.e., the number of groups causing more systematic inaccuracy in the estimation of the number of dots than vice versa). In contrast, parallel organization should be characterized by equal interference of one enumeration process with the other. Estimation should be most accurate for congruent grouped arrays (i.e., 6 groups with 6 dots each) and equally inaccurate for incongruent arrays because either number interferes with the enumeration of the other. We would expect a Stroop-like pattern of congruency and interference effects between the two numbers (MacLeod, 1991, 2010).

## This study

We tested whether dots and groups in grouped arrays can be enumerated quickly and accurately through subitizing and, if so, whether these multiple subitizing processes happen sequentially, with either dots or groups being prioritized, or in parallel. We used a similar protocol to Friedenberg and Limratana (2005), but limited enumeration to the subitizing range and required participants to enumerate both the number of dots and groups in each trial. Participants were shown a target (digit 3 or 4) followed by a briefly displayed grouped dot array similar to those used in groupitizing studies (Anobile et al., 2020; Ciccione & Dehaene, 2020; Moscoso et al., 2020; Starkey & McCandliss, 2014). To ensure that participants enumerated both the number of dots and groups in each trial, they had to indicate whether the grouped dot array matched the target on either the number of dots or the number of groups in each trial. We measured the match-to-target accuracy for arrays with single dots (1 dot per group) and grouped arrays (2, 3 or 4 dots per group). To ensure that we measured whether participants could use subitizing for enumeration, we only presented the grouped arrays for 150 ms, and immediately masked it afterwards.

We asked two main questions in this study:

1. Does subitizing occur for dots and groups in grouped arrays?
  If both dots and groups in grouped arrays are subitized, we would expect accuracy on the match-to-target task to be high. Further, we would expect no, or only minimal, differences in subitizing performance between arrays with single dots and grouped arrays.
2. Are dots and groups in grouped arrays subitized sequentially or in parallel?

We constructed congruent and incongruent arrays with 3 or 4 dots and groups. We analyzed systematic patterns of interference depending on whether the number of dots and the number of groups were congruent or incongruent to each other and in relation to the target of a given trial (e.g., an array with 4 groups of 4 dots is congruent when presented with the target 4, an array of 4 groups of 3 dots is incongruent—it has incongruent groups when presented with the target 3 and incongruent dots when presented with the target 4). If dots and groups were subitized in parallel, we would expect that participants would be most accurate for congruent arrays and would expect equal accuracy for arrays with incongruent dots and arrays with incongruent groups. In contrast, we would expect a difference in accuracy between incongruent arrays if subitizing took place sequentially.

## Methods

This study was preregistered on the Open Science Framework (https://osf.io/68xzs). All data and materials are available (https://osf.io/hc8r7/).

### Participants

We recruited 22 participants from a pool of students and staff at Loughborough University. Two participants were excluded and replaced according to preregistered criteria (one did not complete the experiment and one had an overall task accuracy below 60%).

Our final sample consisted of the preregistered sample size of 20 adults (age in years: *M* = 24.55, *SD* = 6.95, gender: 14 females, six males). All had normal or corrected-to-normal vision and no neurological conditions. Participants were compensated for taking part in the study with £4. The study protocol was approved by Loughborough University’s Ethics Approvals (Human Participants) Subcommittee.

### Materials

**Fig. 1:**
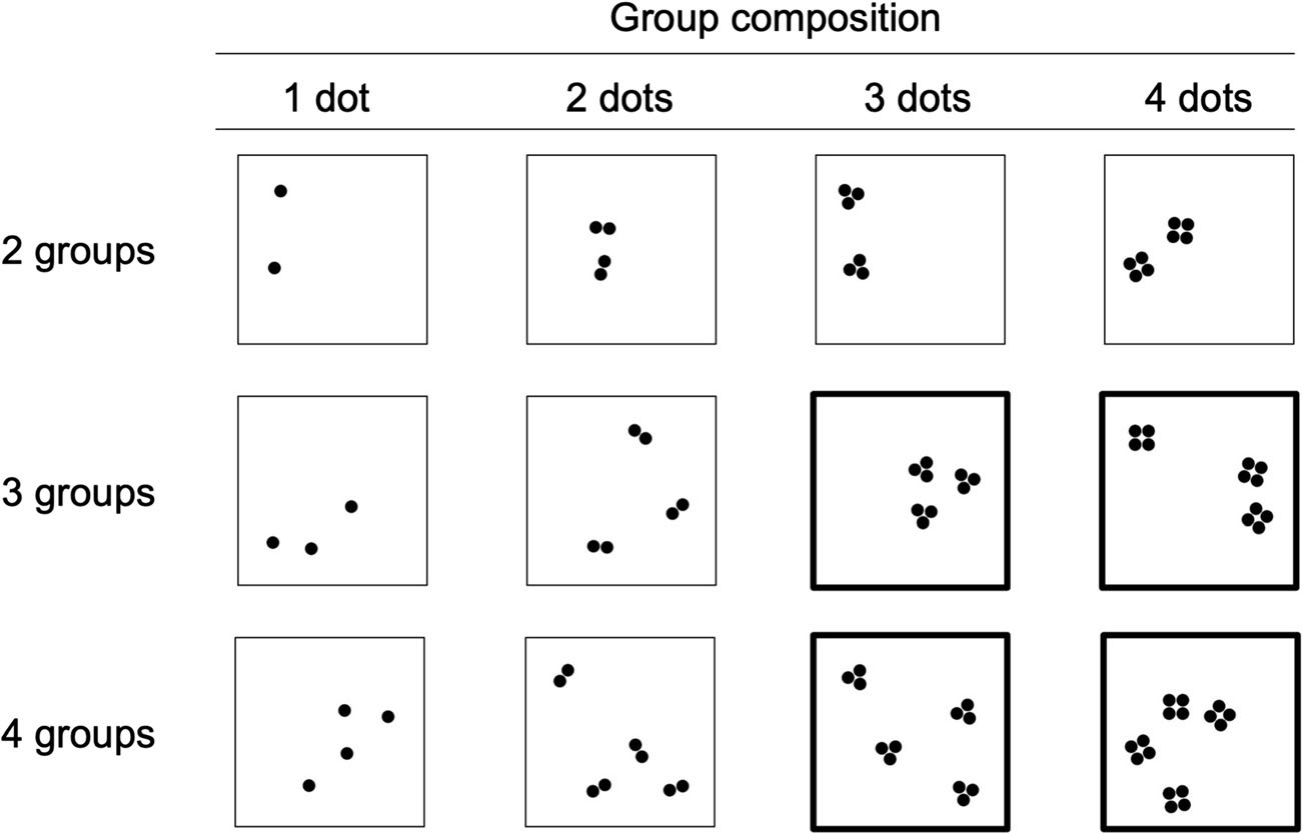
Examples of all types of dot arrays used in this experiment. The combination of different number of groups and different numbers of dots composing each group created 12 different arrays in four group compositions. Bold outline arrays were used in our second analysis and represent the congruent condition (3 groups × 3 dots for Target 3, and 4 groups × 4 dots for Target 4) and the incongruent conditions (3 groups × 4 dots and 4 groups × 3 dots, having incongruent groups or dots, respectively, in relation to either Targets 3 or 4)

We used 720-pxl × 720-pxl large arrays (13.5° of visual angle for participants sitting 80 cm away from the screen) of black dots (0.7° diameter) and dot groups on a grey background to create 12 different compositions of dots and groups in grouped arrays, as shown in Fig. 1. We composed groups of either 1, 2, 3, or 4 dots each with a 1-mm distance between adjacent dots in the groups and a minimum distance of 0.7° between groups. Dots within a group had a distance of 0.7° to the adjacent dot and were arranged symmetrically. Each group composition (1, 2, 3, 4 dots) was used to create arrays with 2, 3, and 4 groups. All groups were symmetrical and groups of the same number of dots were identical to each other. The position of the groups was determined randomly in a 4 × 4 grid with a jitter of 3.6° to avoid alignment. Four random arrays were created for each number of groups with each group composition. Hence, a total number of 48 unique dot arrays were used in this experiment.

### Procedure

The experiment took place in a small room with controlled lighting conditions. Participants completed the task individually. They sat approx. 80 cm in front of a 17-in. laptop on which the experiment was presented in an otherwise stimulus deprived room. PsychoPy software (Version 1.85.6) was used to present stimuli and log participant responses (Peirce, 2009).

**Fig. 2:**
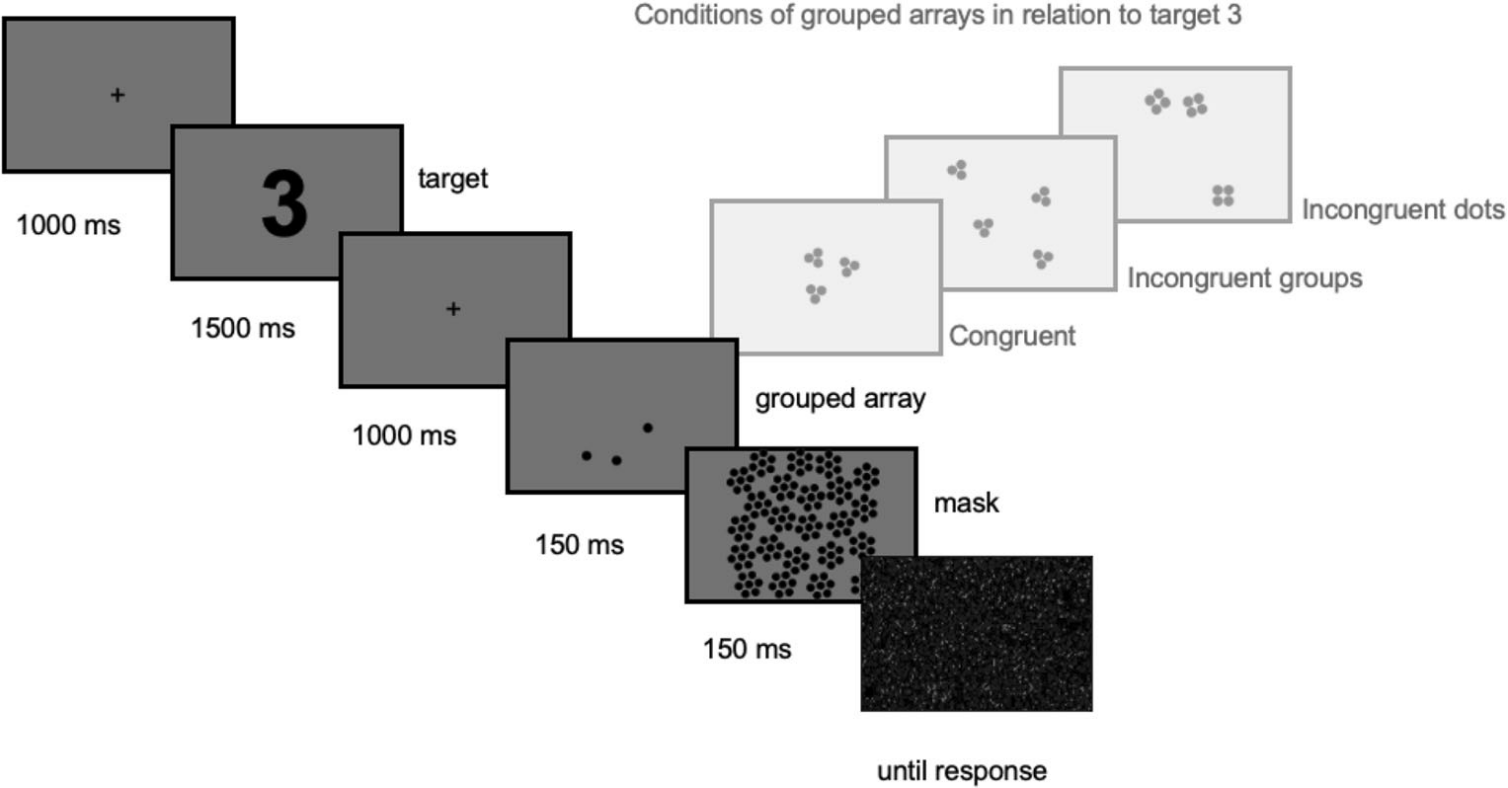
Illustration of the procedure for each experimental trial. Participants were asked to indicate whether the grouped array matched the previously presented target on either the number of dots per group or the number of groups. The next trial started after participants responded. The relation between the grouped array and the target of the trial determined the condition. In congruent grouped arrays both the number of dots per group and the number of groups matched the target. In grouped arrays with incongruent groups only the number of dots per group matched the target and in grouped arrays with incongruent dots only the number of dots matched the target

Participants were instructed to indicate for each trial whether either the number of groups or the number of dots per group in a grouped dot array matched the target digit. Figure 2 depicts the routine of each trial. The beginning of a trial was indicated by a fixation cross appearing in the center of the screen for 1,000 ms. This was followed by the target. The target was the Arabic digit 3 or 4 presented in the center of the screen for 1,500 ms. After the target, participants were again shown a fixation cross for 1,000 ms. The array of dots groups was then flashed for 150 ms and masked with dots covering the full array for again 150 ms. At the end of the trial participants would see a mask until they responded. Participants were instructed to remember the target and evaluate for the following flashing array if either the number of dots per group or the number of groups matched the target or if neither matched the target. Responses were made on a standard UK keyboard using the letters *s* and *k* covered with a red and green sticker to indicate either a match between target and array, or no match. Before the experiment participants were shown instructions including examples of matching and nonmatching trials for the target digit 2. The sheet with the examples was also available to the participants throughout the experiment for reference. For this reason, the target digit 2 was not used in the experiment.

The 48 different dot arrays were each presented with both target digits, resulting in 96 unique trials. Each unique trial was presented 4 times while the dot array was changed by assigning a random orientation (0°, 90°, 180°, 270°) in each trial. The order of the resulting total 384 trials was randomized. Arrays matched the target in 50% of all trials. Trials were presented in 12 blocks, which allowed participants to take breaks between blocks as required.

### Analyses

We used JASP (JASP Team, 2018) and RStudio (RStudio Team, 2020) running R 3.6.2 (R Core Team, 2019) for data analysis. We used Bayesian inference for our analysis, because quantifying evidence in favor of both difference and equality was crucial to testing our hypotheses (Wagenmakers et al., 2018). We report Bayes factors in favor of the alternative (BF_10_). A BF_10_ larger than 1 indicates evidence supporting the alternative, and a BF_10_ less than 1 indicates evidence for the null. We also report 95% credible intervals (CI_95%_ ) around mean estimates.

## Results

**Fig. 3:**
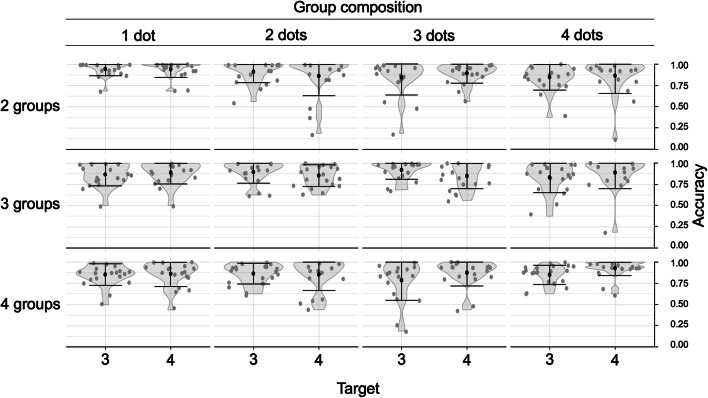
Raw accuracy data for trials of compositions of dots and groups presented with each target digit. Grey points represent individual participants. Black points and error bars represent Mean ± 1 *SD*

As shown in Fig. 3, overall task accuracy was high (*M* = .876, *SD* = .106, min = .617, max = .982, CI_95%_ [.826, .925]). Subitizing overall occurred successfully across all different compositions of dots and groups and both target digits.

### Does subitizing occur for dots and groups in grouped arrays?

**Fig. 4:**
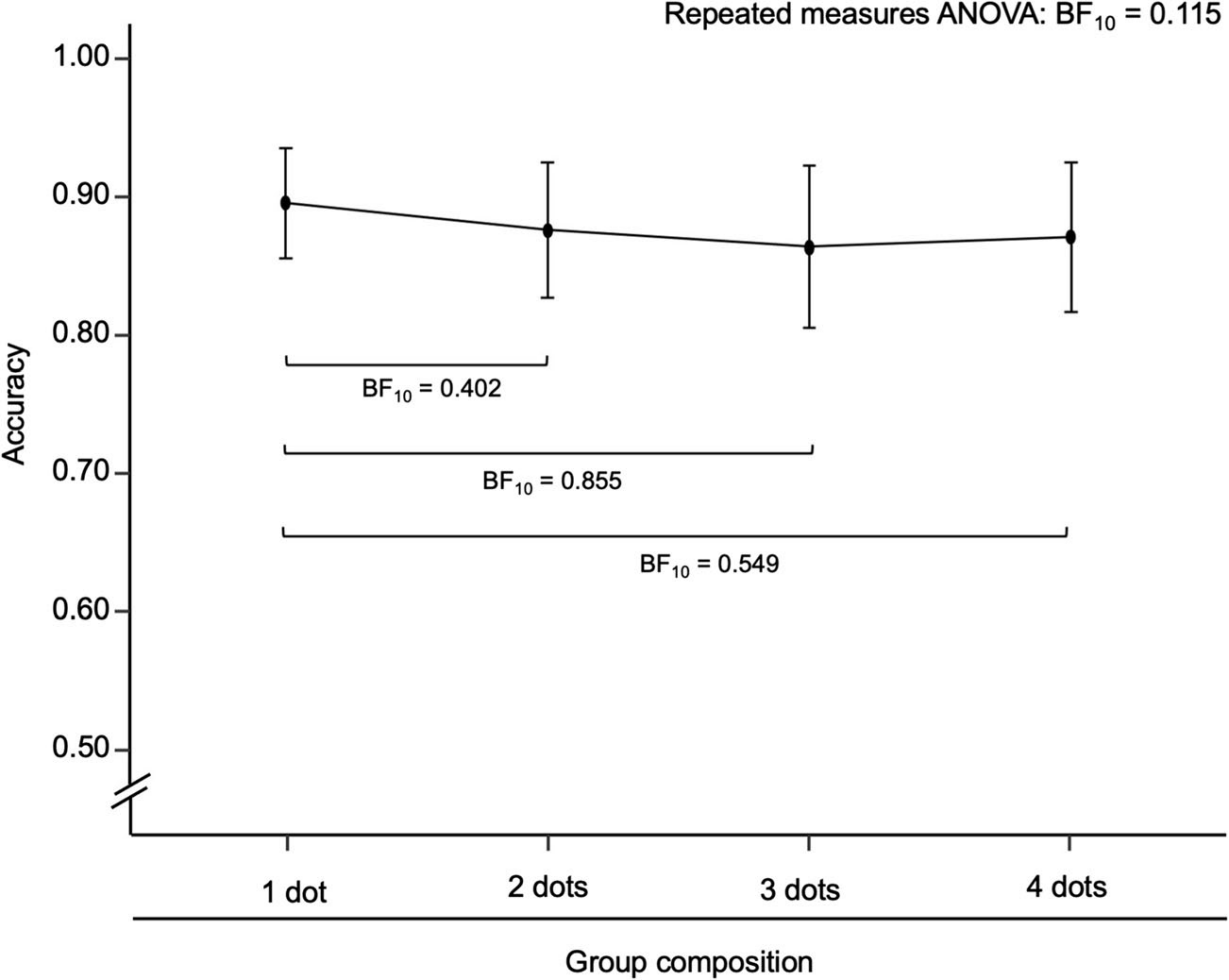
Effect of group composition on task accuracy. Black points and error bars represent Mean ± CI_95%_

As shown in Fig. 4, mean match-to-target accuracy was similar for arrays with groups composed of 1 dot, effectively being ungrouped arrays and grouped arrays with groups composed of 2 dots, 3 dots, and 4 dots. In order to test whether subitizing occurred with equal success for dots and groups, we ran a series of preregistered paired Bayesian *t* tests^1^ comparing the mean match-to-target accuracy of arrays with groups composed of 1 dot (i.e., ungrouped arrays) to grouped arrays with groups composed of 2, 3, and 4 dots, respectively. Each of those mean accuracies was taken from 96 trials, out of which half were presented with the target 3 and the other half with the target 4. We used uninformed Cauchy priors (*r* = .707) to calculate Bayes factors for each *t* test. The results, shown in Fig. 4, were inconclusive, with individual Bayes factors not indicating evidence for or against a difference between accuracies.

After data collection, we decided to follow up on these analyses with a 4 × 2 Bayesian repeated-measures analysis of variance (ANOVA) that included group composition and target (3 vs. 4) as a factor. This decision made analyses consistent with the preregistered analysis for our second research question. The results again favored a null model over the full model (BF_10_ = 0.033). Considering that by design the group compositions with 1 dot or 2 dots included fewer trials that matched the target digit, we also followed up with a model that included target match versus nonmatch as a factor. Again, we found strong evidence in favor of a null model (BF_10_ = 0.031). Overall, there was no evidence for a difference in the subitizing accuracy between ungrouped arrays and grouped arrays, indicating that subitizing occurred successfully for both dots and groups in grouped arrays.

### Are dots and groups in grouped arrays subitized in parallel or sequentially?

**Fig. 5:**
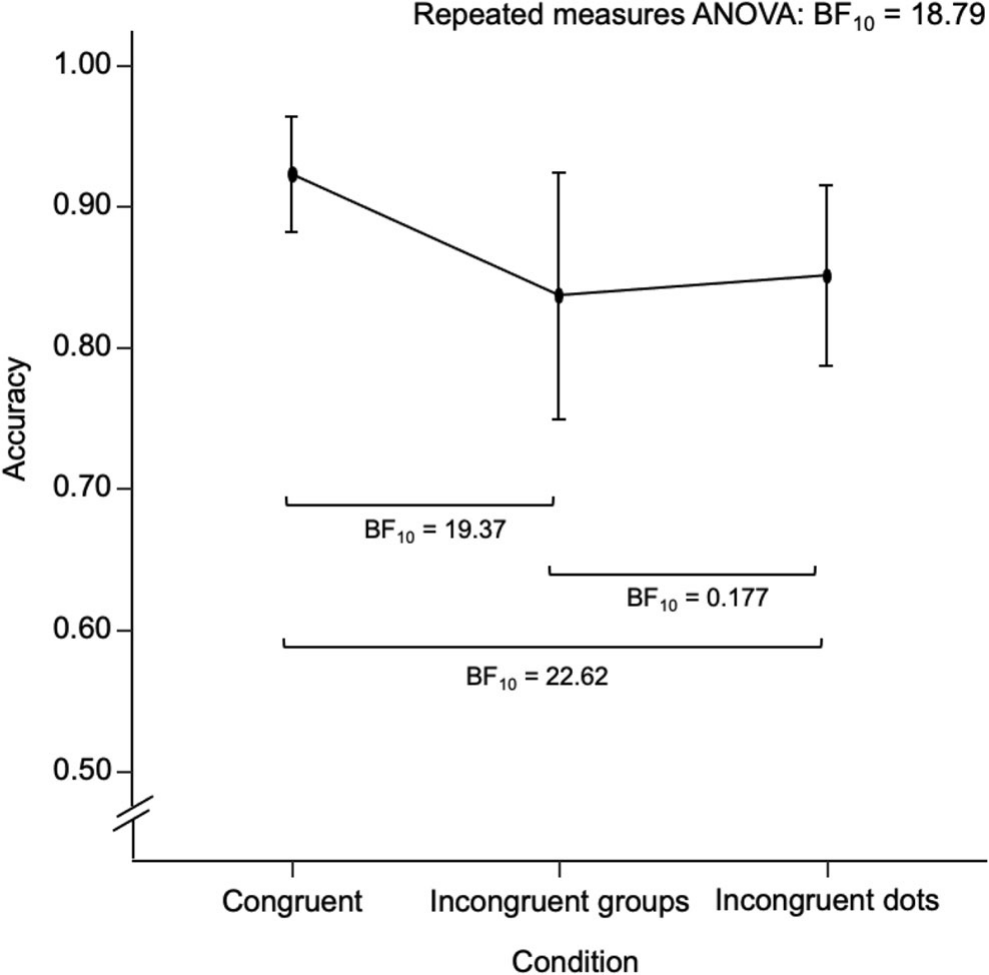
Effect of condition on task accuracy. Conditions are defined by the congruency of the number of dots and the number of groups to each other and in relation to the Targets 3 and 4. Black points and error bars represent Mean ± CI_95%_

We modelled mean match-to-target accuracy between the three conditions of dot arrays, defined by their congruency relative to the targets 3 and 4 (bold in Fig. 1, examples in Fig. 2). Each of these conditions contained 32 trials. Mean match-to-target accuracy was highest for congruent arrays which contained the same number of dots and groups compared with arrays with incongruent groups and arrays with incongruent dots (see Fig. 5). The opposing predictions of sequential versus parallel subitizing of dots and groups were examined with a Bayesian repeated-measures ANOVA. We tested the main effects of condition (congruent vs. incongruent groups vs. incongruent dots), the main effect of the target (3 vs. 4) and the interaction between these factors for inclusion in the model. We found very strong evidence favoring a model including both main effects and no interaction against a null model (*R*^2^ = .561, BF_10_ = 67.852). There was strong evidence for the main effect of condition (η^2^ = .279, BF_10_ = 18.785).

Following up on the main effect of condition, we found strong evidence for higher mean accuracy in the congruent condition compared with both the condition with incongruent groups and the condition with incongruent dots. We found moderate evidence for the equality between the condition with incongruent groups and the condition with incongruent dots, suggesting there was no systematic performance advantage for either condition^2^(see Fig. 5).

## Discussion

### Summary of main findings

We investigated subitizing of dots and groups in grouped arrays, and addressed two main questions: (i) Does subitizing occur for dots and groups in grouped arrays? (ii) Are dots and groups in grouped arrays subitized in parallel or sequentially? Subitizing was measured in a match-to-target task in which participants had to indicate whether the number of dots and/or groups in a briefly flashed array matched the target. Mean match-to-target accuracy was not different between arrays with groups composed of a single dot compared with any dot arrays with groups composed of a subitizable number of dots. We concluded that subitizing can successfully occur for dots and groups in grouped arrays and is not substantially impacted when compared with subitizing an array of single dots.

We further analyzed how the subitizing processes for dots and groups in grouped arrays are organized. We tested two possibilities with conflicting predictions: sequential organization and parallel organization. We analyzed interference on the match-to-target accuracy between arrays with a congruent number of dots and groups and arrays with an incongruent number of dots and groups. We found that incongruent dots interfere with the subitizing of groups to a similar extent as how incongruent groups interfere with the subitizing of dots. These results suggest that neither dots nor groups were prioritized for subitizing. Our data imply that multiple subitizing processes can occur in parallel, and that dots and groups in grouped arrays can be subitized with equal priority.

### Groupitizing mechanisms

There are at least three areas of consideration to understand groupitizing mechanisms: how the array is grouped, how numerical information is extracted, and how this numerical information is used to enumerate the total number of items. Our study provides first evidence for parallel subitizing of dots and groups as a way of extracting numerical information from grouped arrays. We discuss what these findings imply for theories on the strategic use of arithmetic abilities to quickly enumerate the total number of dots and how these implications rely on how the array is grouped.

Groupitizing mechanisms are a matter of debate. Starkey and McCandliss (2014) found that groupitizing was positively associated with children’s arithmetic abilities, an association also shown in adults (Anobile et al., 2020; Ciccione & Dehaene, 2020; Moscoso et al., 2020). Proposed mechanisms for the association between groupitizing and arithmetic abilities include the extension of the subitizing range through arithmetic abilities (Moscoso et al., 2020), the use of fast repeated addition (Wender & Rothkegel, 2000), conceptual knowledge about the composition of large numbers from small numbers (Sarama & Clements, 2009; Starkey & McCandliss, 2014; Wästerlid, 2020), and the use of mental multiplication (Ciccione & Dehaene, 2020). All of these proposed mechanisms concern how numerical information is used to quickly enumerate the total number of items in the array. They do not provide an explanation for how the numerical information needed for a mental calculation is extracted from grouped arrays in the first place. Our findings offer some insight into this issue by providing evidence that operands for mental calculations can be found via subitizing.

Our study suggests that the parallel subitizing of dots and groups in grouped arrays may provide the enumeration processes that are necessary for groupitizing via mental multiplication. Ciccione and Dehaene (2020) proposed that visually grouping dots into identical groups allowed participants to use the number of groups and dots per group as operands in the retrieval of multiplication facts (such as 4 × 3 = 12). Multiplication facts are highly trained and can be retrieved from memory very fast and accurately (Campbell & Graham, 1985). Grouped arrays with identical groups can be ‘groupitized’ within 1,000–1,500 ms (Ciccione & Dehaene, 2020), but the retrieval of multiplication facts alone takes roughly 1,000 ms (Verguts & Fias, 2005). Groupitizing via mental multiplication therefore requires a particular way of extracting numerical information from grouped arrays: it requires an enumeration process that quickly and accurately (i) enumerates the number of dots per group and (ii) enumerates the number of groups in a grouped array. In our study, we found that not only can subitizing be used to enumerate dots and groups within a fraction of a second but also that these two subitizing processes happen in parallel and are able to extract numerical information on dots and groups quickly, accurately, and with equal priority. Evidence for the parallel subitizing of dots and groups further supports mental multiplication as a groupitizing mechanism and offers an additional account for why groupitizing is most efficient for arrays grouped into a subitizable number of identical groups with a subitizable number of dots.

Parallel subitizing of dots and groups can also help us understand how the way that arrays are visually grouped affects the success of groupitizing. People tend to make very similar and predictable judgements about what constitutes a group in an array of dots, and they actively look for these groupings when enumerating (Im et al., 2016) different forms of visual grouping can affect how accurately a large set of items can be enumerated beyond just the groupitizing literature (Chesney & Gelman, 2012; Franconeri et al., 2009; He et al., 2009, Trick & Enns, 1997). For groupitizing, enumeration accuracy seems to be most strongly improved by grouping arrays using visual proximity, and by grouping them into a subitizable number of identical groups with a subitizable number of dots (Anobile et al., 2020; Ciccione & Dehaene, 2020; Moscoso et al., 2020; Starkey & McCandliss, 2014; Wender & Rothkegel, 2000). Our findings suggest the likely reason is that this particular way of visual grouping supports parallel subitizing of dots and groups, which in turn provides operands for mental calculations. The groupitizing effect can also be found if arrays are grouped by temporal proximity or separated colors (Anobile et al., 2020; Ciccione & Dehaene, 2020), but seems to be heavily impaired when items are grouped by shape or color, but interspersed (Liu et al., 2020; Watson et al., 2005). A possible reason is that items that belong to a spatially connected group can be easily subitized independently of visual proximity or grouping features such as shape, but subitizing performance is impaired if items belong to several spatially separate groups (Poncet & Chakravarthi, 2021). Although not yet present in the groupitizing literature, arrays could also be grouped by other forms of visual similarity, continuity, or even common movement (Poom et al., 2019; Razpurker-Apfeld & Kimchi, 2007; Todorovic, 2008; Wagemans et al., 2012). The findings of our study prompt us to ask whether the differences in groupitizing accuracy between different forms of visual grouping could be explained by the extent to which these forms of grouping support parallel subitizing of dots and groups.

We can only speculate about the mechanisms that enable the parallel subitizing of dots and groups. Our definition of parallelism relied on the enumeration of dots interfering equally with the enumeration of groups and vice versa. This includes the possibility of subitizing processes happening in extremely fast succession, but also simultaneously or with alternating priority across trials. It is further possible that subitizing of dots and groups as observed in our study was supported by particular characteristics of our experimental setup—for example, the instruction to attend to both the number of dots and groups or the particular arrangement in the arrays we used. Dot arrays contained groups of dots that were not arranged randomly but in identical and canonical shapes with close visual proximity. Such visual groups are very easily perceived and indexed as visual entities (Trick & Enns, 1997; Kimchi, 2009; Kimchi & Razpurker-Apfeld, 2004). Subitizing of the number of groups might therefore have been supported by this indexing of entities on a visual map (Trick, 1989; Trick & Pylyshyn, 1994). In contrast, subitizing the number of dots in each group might have been supported by the association between the groups’ canonical shapes and number (i.e., three dots being always arranged in a triangle; Allen & McGeorge, 2008; Mandler & Shebo, 1982; Wender & Rothkegel, 2000). Subitizing via shape association is not limited by the same subitizing range of around four items as subitizing via visual indexing (Wender & Rothkegel, 2000; Wolters et al., 1987). Probing the limits of parallel subitizing might allow insight into how the groupitizing effect is shaped by a combination of mechanisms for extracting numerical information with strategies of using this numerical information.

The groupitizing effect has been established to rely on how visual grouping can allow the use of mental calculations as effective enumeration strategies. Establishing the capacity for parallel subitizing of dots and groups in grouped arrays contributes to our effort to untangle the mechanisms behind groupitizing. The results of this study suggest that groupitizing is not only a process of enumeration with subitizing-like characteristics, but, under the right conditions, likely rooted in subitizing processes unique to grouped arrays.

## Footnotes

1 In the preregistration, we mentioned controlling those Bayes factors for multiple comparisons. However, this control is only conservative in terms of finding evidence for a difference, but makes a test more liberal in terms of finding evidence for the null. Because we also wanted to conservatively test the null, we decided against controlling Bayes factors for multiple comparisons after data collection. Indeed, the now-inconclusive Bayes factors in our analyses would have all been interpreted as moderate evidence for the null if we had applied the control as preregistered.

2 To test the robustness of this analysis, we also calculated it after removing outliers. Two participants performed at accuracies below 50% in either the incongruent groups or incongruent dots condition. Bayes factors supporting the difference between the congruent and each incongruent condition decreased to indicating only moderate evidence (BF_10_ = 4.47 and BF_10_ = 11.281). The Bayes factor indicating moderate evidence for the equality between incongruent conditions did not change substantially (BF_10_ = 0.249). All conclusions from the preregistered analysis remained supported.
